# Sleep quality, neurocognitive performance, and memory self-appraisal in middle-aged and older adults with memory complaints

**DOI:** 10.1017/S1041610220003324

**Published:** 2020-09-28

**Authors:** Prabha Siddarth, Kitikan Thana-udom, Rashi Ojha, David Merrill, Joseph M. Dzierzewski, Karen Miller, Gary W. Small, Linda Ercoli

**Affiliations:** 1 Semel Institute for Neuroscience and Human Behavior, UCLA, Los Angeles, CA, USA; 2 Longevity Center, David Geffen School of Medicine, UCLA, Los Angeles, CA, USA; 3 Division of Geriatric Psychiatry, Department of Psychiatry and Biobehavioral Sciences, UCLA, Los Angeles, CA, USA; 4 Department of Psychiatry, Faculty of Medicine Siriraj Hospital, Mahidol University, Bangkok, Thailand; 5 Pacific Brain Health Center at Pacific Neuroscience Institute, Santa Monica, CA, USA; 6 Department of Psychology, Virginia Commonwealth University, Richmond, VA, USA

**Keywords:** sleep quality, age-related cognitive decline, memory complaints

## Abstract

**Objective:**

Because of inconsistent findings regarding the relationship between sleep quality and cognitive function in people with age-related memory complaints, we examined how self-reports of sleep quality were related to multiple domains of both objective and subjective cognitive function in middle-aged and older adults.

**Design:**

A cross-sectional study involving analysis of baseline data, collected as part of a clinical trial.

**Measurements:**

Two hundred and three participants (mean age = 60.4 [6.5] years, 69.0% female) with mild memory complaints were asked to rate their sleep quality using the Pittsburgh Sleep Quality Index (PSQI) and their memory performance using the Memory Functioning Questionnaire (MFQ), which measures self-awareness of memory ability. Neurocognitive performance was evaluated using the Continuous Performance Test (CPT), Trail Making Test, Buschke Selective Reminding Test, and the Brief Visuospatial Test – Revised (BVMT-R).

**Results:**

Total PSQI scores were significantly associated with objective measures of sustained attention (CPT hit reaction time by block and standard error by block) and subjective memory loss (MFQ frequency and seriousness of forgetting). The PSQI components of (poorer) sleep quality and (greater) sleep disturbance were related to (worse) sustained attention scores while increased sleep latency and daytime sleepiness were associated with greater frequency and seriousness of forgetting.

**Conclusions:**

Sleep quality is related to both objective measures of sustained attention and self-awareness of memory decline. These findings suggest that interventions for improving sleep quality may contribute not only to improving the ability to focus on a particular task but also in reducing memory complaints in middle-aged and older adults.

## Introduction

Considerable evidence indicates the benefits of sufficient and high-quality sleep for physical and mental health, including cognition. Although the nuances of sleep patterns may change during a person’s lifetime (Ohayon *et al*., 2004), sleep remains critical for well-being at all stages of life. Some of the changes in sleep patterns that can occur in older adults include a reduction in total sleep time, decreased sleep efficiency, decreased quantity of slow wave and REM sleep, increased sleep fragmentation, and increased awakenings after sleep onset (Campbell and Murphy, 2007; Dijk *et al*., 2001; Dorffner *et al*., 2015; Ohayon *et al*., 2004; Scullin and Bliwise, 2015; Wolkove *et al*., 2007). Further, older adults report worse sleep quality compared to younger people (Crowley, 2011).

In addition to changes in sleep patterns, aging is also associated with changes in cognition, which can include a decline in memory, information processing speed, attention and executive function (Davis *et al*., 2013; Salthouse, 2010), and both the quantity and quality of sleep may be a moderator for cognitive aging (Loerbroks *et al*., 2010). Longitudinal data show that adequate and high-quality sleep promotes cognitive health (Dzierzewski *et al*., 2018; Vance 2012), while increased sleep fragmentation and sleep disruption in older adults is associated with a higher rate of cognitive decline over a 3- to 5-year follow-up (Blackwell *et al*., 2014; Lim *et al*., 2013; McSorley *et al*., 2019).

Other studies that have examined the relationship between sleep problems and cognitive function in older adults have yielded inconsistent results. Some cross-sectional studies have not shown associations between subjective sleep quality and global cognition (Saint Martin *et al*., 2012) or any domains of cognition (Blackwell *et al*., 2011; Miyake *et al*., 2000), whereas other cross-sectional investigations have indicated an association between low-quality sleep and worsening of at least one component of cognitive performance, such as decline in processing speed (Bastien *et al*., 2003), decreased executive function (Nebes *et al*., 2009; Waters and Bucks, 2011), and impaired episodic memory (Tsapanou *et al*., 2017). Such inconsistencies may be attributed to differences in controlled variables, such as depressive symptoms, anxiety, or sleep medication use, or the inclusion of heterogeneous populations across studies, such as the inclusion of neurological and psychiatric comorbidities in participants (Bastien *et al*., 2003; Foley *et al*., 2003; Moore *et al*., 2001). Sample heterogeneity is particularly relevant because each domain of cognitive function has an intricate set of multiple sub-processes (Glisky, 2007) that may be uniquely linked to sleep quality in different populations. The lack of a uniform set of cognitive measures across studies makes it challenging to compare findings. In addition to the objective measures of cognition, subjective memory complaints, which can occur in the absence of objective cognitive decline, may predict future cognitive impairment and dementia (Choe *et al*., 2018; Jessen, 2010). Only a few studies, however, have examined the link between sleep quality and subjective memory complaints (Gamaldo *et al*., 2019; Kang *et al*., 2017), and these have indicated a possible connection between poor sleep and memory dysfunction.

A few imaging studies have investigated the effect of sleep quality on alterations in brain structure in older adults. In adults 55 years and older, self-reported short sleep duration was associated with greater age-related brain atrophy and cognitive decline 2 years later (Lo *et al*., 2014). Worse self-reported sleep was also found to be associated with greater hippocampal volume reduction across the adult lifespan (Fjell *et al*., 2020); however, the effect sizes (ES) were small. Thus, an understanding of how sleep quality may affect brain structure and cognition remains limited.

Given the lack of consistent findings in the literature with respect to sleep quality and neurocognitive performance and the paucity of research with respect to sleep quality and subjective memory complaints, we examined the association between subjective sleep measures and specific aspects of neurocognitive performance and self-reported memory in non-demented middle-aged and older adults with subjective memory complaints. We investigated four domains of objective cognition – simple attention, sustained attention, memory recall, and executive function, and two aspects of subjective memory performance – frequency of forgetting and seriousness of forgetting. We anticipated that sustained attention, memory recall, executive function, and the two self-report memory measures would show significant impairment with poorer sleep quality, while simple attention was less likely to be affected.

## Methods

### Participants

The present study is a secondary analysis of baseline data collected on 221 middle-aged and older adults, who participated in a randomized, placebo-controlled trial exploring the cognitive effects of daily consumption of pomegranate juice (Identifier: NCT02093130; Siddarth *et al*., 2020) approved by the University of California, Los Angeles, Institutional Review Board. Participants were recruited through advertisements, media, and referrals from physicians and families. Our study protocol detailed the methods and procedures, as well as pre-specified inclusion and exclusion criteria. Exclusion criteria included participants with a diagnosis of Alzheimer’s disease or other dementia, a major psychiatric or neurological disorder (e.g. major depressive disorder, schizophrenia, Parkinson’s disease, Huntington’s disease), alcohol or substance abuse, or a history of head trauma or systemic disease affecting brain function (e.g. systemic lupus erythematosus, congestive heart failure). Inclusion criteria were: (1) subjective memory complaints (initially assessed during a phone screen by an affirmative response to the question, “Are you now experiencing or have you experienced memory problems over the past 6 months?” and confirmed by responses to the Memory Functioning Questionnaire [MFQ]); (2) aged 50 or greater; (3) no significant cerebrovascular disease defined as Stroke Risk Factor Prediction < 4 (Wolf *et al*., 1991); (4) normal cognition, defined as Montreal Cognitive Assessment (MOCA) ≥ 25 (Nasreddine *et al*., 2005); and (5) absence of significant depressive symptomology, defined as Beck Depression Inventory-II (BDI-II) < 18 (Beck *et al*., 1996). Participants underwent neuropsycho-logical and laboratory testing, including APOE genotyping. Written informed consent was obtained from all subjects in accordance with the University of California, Los Angeles Human Subjects Protection Committee procedures. The trial began in January 2014 and was completed in May 2018, and all data were collected at the Semel Institute for Neuroscience and Human Behavior and Center for Human Nutrition, David Geffen School of Medicine at the University of California, Los Angeles, CA. All data used in the present study were collected at baseline before the intervention was begun.

### Measures

#### Sleep quality

Sleep data were obtained using a self-report questionnaire, the Pittsburgh Sleep Quality Index (PSQI). The PSQI assesses sleep quality exploring seven components of sleep. Each item has a range of 0 to −3, and the seven items are summed for a Total PSQI score (lower scores indicating better sleep quality). With a cut-off score of 5, a PSQI total has a sensitivity of 89.6% and specificity of 86.5% for identifying disordered sleep (Buysse *et al*., 1989). The Total PSQI score was used as a continuous variable in analyses. In addition, we also examined the seven component scores. The distribution of these item scores did not allow their use as continuous variables in analyses, and hence they were dichotomized as below, depending on their distribution, such that there were sufficient participants in each group (see Table 1):

1. Subjective sleep quality: Good (score 0–1) versus bad (score 2–3);
2. Sleep latency: Less than or equal to 15 minutes (score 0) versus more than 15 minutes (score 1–3);
3. Sleep duration: More than 7 hours (score 0) versus 7 hours or less (score 1–3);
4. Habitual sleep efficiency: more than 85% (score 0) versus 85% or less (score 1–3);
5. Sleep disturbance (i.e. sleep fragmentation or sleep disruption): Less disturbed (score 0–1) versus more disturbed (score 2–3);
6. Use of sleeping medication: No use (score 0) versuss use (score 1–3);
7. Daytime dysfunction: No problem (score 0) versus problem with daytime dysfunction (score 1–3).

#### Obstructive sleep apnea (OSA)

In addition to the PSQI, the STOP-BANG questionnaire (Chung *et al*., 2016) was used to screen for OSA. This instrument assesses four subjective items and four clinical items: S – “Do you snore loudly?”; T – “Do you often feel tired, fatigued, or sleepy during daytime?”; O – “Has anyone observed you stop breathing during your sleep?’; and P – “Do you have or are you being treated for high blood pressure?” The BANG part of the questionnaire consists of four demographic questions associated with risk for OSA: B – increased body mass index (over 35 kg/m2); A – aged over 50 years; N – neck circumference over 40 cm; and G – male gender. For each question, answering “yes” scores 1, while a “no” response scores 0, resulting in a range of 0–8. A standard cut-off score of 3 is usually taken to indicate high risk for OSA.

#### objective measures of cognitive functioning

Within 1 month of the PSQI assessment, participants underwent a full neuropsychological assessment that focused on four domains: (1) **Simple attention**: measured using the five T-scores of the Continuous Performance Test (CPT) (Conners, 2000): detectability, hit reaction time, standard error, reaction time by interstimulus interval, and standard error by interstimulus interval; (2) **Sustained attention** (ability to maintain focus on a specific task for an extended period of time) (Fortenbaugh *et al*., 2017) measured by hit reaction time by block and standard error by block (Conners, 2000); (3) **Executive function**: this domain included two major components of executive function, namely cognitive set-shifting and the ability to inhibit responses. Set-shifting ability (adjusted for processing speed) was measured by subtracting the Trail Making Test A time score from Trail Making Test B time score (Arbuthnott and Frank, 2000). Inhibition was measured using T-scores of commission errors, and hit reaction time of CPT; high T-scores for both commission errors and hit reaction times are indicators of impulsivity (Conners, 2000). and (4) **Recall memory:** measured using the delayed recall score of Buschke–Fuld Selective Reminding Test (SRT) (Buschke, 1973) and the delayed recall score of the Brief Visual Memory Test – Revised (BVMT-R) (Benedict *et al*., 1996). T-scores and raw scores were converted to z-scores using the overall sample mean and standard deviation. For variables in which better performance was represented by lower values (e.g. T-scores of CPT, timed scores of the Trail Making Test), z-scores were reversed so that a higher score indicated better performance. A domain z-score was obtained by averaging z-scores.

#### Subjective assessment of memory functioning

Participants completed MFQ (Gilewski *et al*., 1990); two factor scores were used in the analysis from this 64-item questionnaire: frequency of forgetting (MFQ1) and seriousness of forgetting (MFQ2). Higher scores reflect higher levels of self-perceived memory functioning. Factor structure is stable across age groups and internal consistency is high, with Cronbach’s alpha values for its factor scores ranging from 0.83 to 0.94 (Zelinski *et al.*, 1990). The MFQ shows moderate concurrent validity with objective memory performance (Zelinski *et al*., 1990).

### Data analysis

Prior to analyses, all data were screened for outliers and normality assumptions. Baseline clinical characteristics were described using total number (percent) for categorical data and mean (standard deviation) for continuous data. A separate general linear model was estimated to examine if sleep quality measured by Total PSQI was significantly associated with objective cognitive performance in each of the four domains, controlling for age, gender, education level, and baseline MOCA score. We also examined whether age, gender, OSA risk, and APOE-4 status moderated the relationship between cognitive functioning and sleep, by including the interactions in turn (age × PSQI; gender × PSQI; OSA risk × PSQI, ApoE4 × PSQI) in the models. The moderating effects of age, gender, OSA risk, and ApoE4 were tested since all these variables are known to affect sleep and/or cognitive performance (Gosselin *et al*., 2019; Krishnan and Collop, 2006; Lim *et al*., 2013; Scullin and Bliwise, 2015). Similar general linear models were estimated to examine if sleep quality was associated with MFQ1 and MFQ2, the two subjective memory scores. Finally, for those cognitive domains and subjective memory scores that yielded significant associations with Total PSQI, we analyzed each PSQI component in a separate model to ascertain which specific sleep component, if any, was associated with cognitive performance and subjective memory complaints. Statistical analyses were performed using IBM SPSS Statistics v21. Using Bonferroni correction for multiple comparisons, findings were considered significant at 0.0125 (0.05/4; two-tailed) for the four objective cognitive domain scores and 0.025 (0.05/2; two-tailed) for the two MFQ scores.

## Results

Of 221 participants, 15 were excluded from the analyses because they did not complete neuropsychological testing (n = 7) or lacked PSQI scores (n = 8); 3 participants with outlying values on TMT-B − TMT-A (z-scores of ≥ 4) were also excluded. As a result, the final sample size was 203, with a mean age of 60.4 (SD 6.5). The sample was predominantly Caucasian (69.5%) and included a higher proportion of women (69.0%), with a mean educational level of 16.4 (2.1) years and mean Total PSQI of 4.9 (2.9) (Table 1). Cognitive test scores are presented in Table 2.

### PSQI and cognitive domains

**Figure 1.**
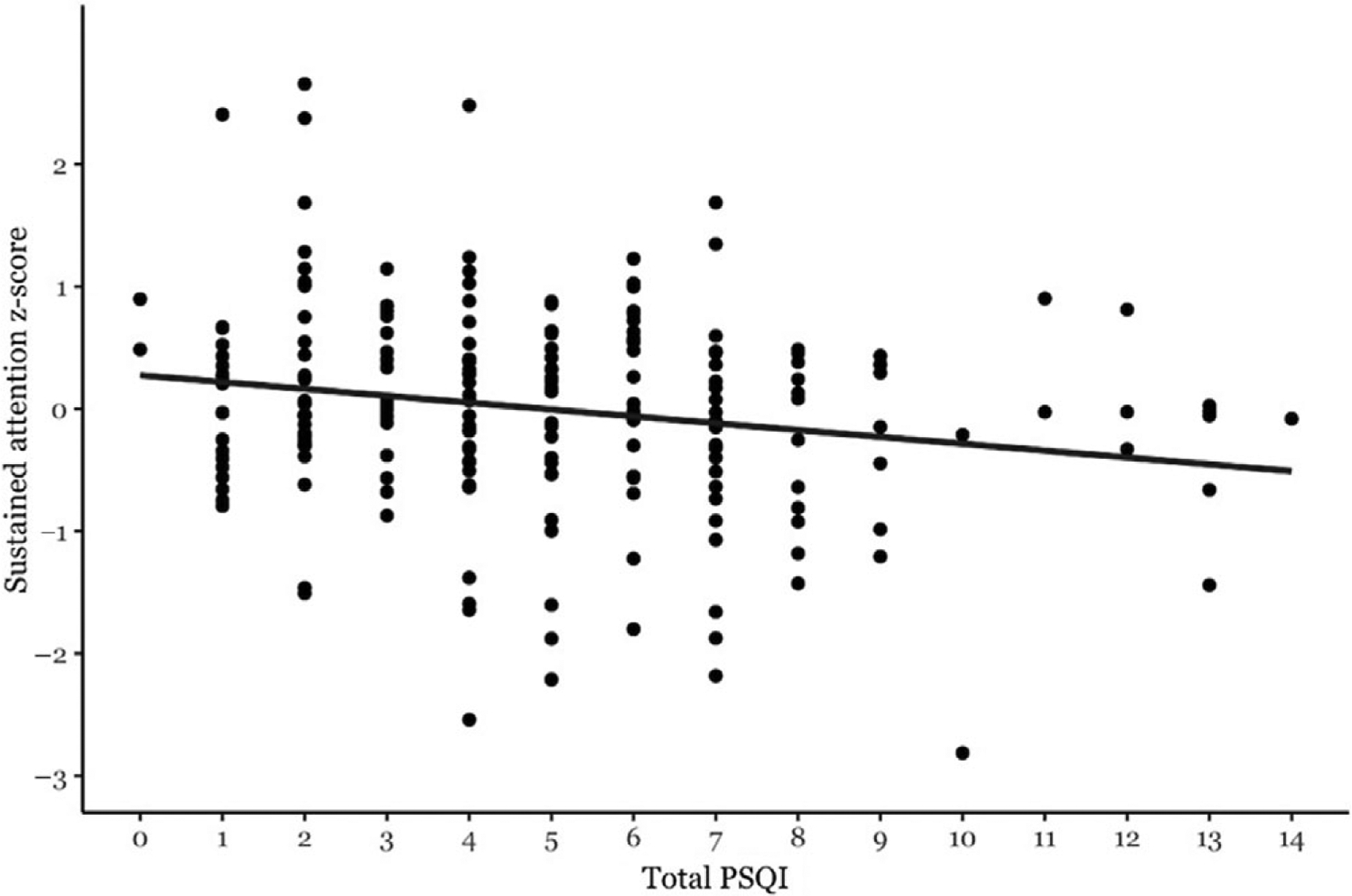
Association between Total PSQI and sustained attention z-score.

Poorer sleep quality, as measured by Total PSQI score, was significantly associated with worse scores on sustained attention [F(1,197) = 10.03, *p* = 0.002, partial r = − 0.22 (Figure 1)]. The Total PSQI score was not significantly associated with simple attention, executive function, or recall memory (Table 3). ApoE4, OSA risk, gender, and age did not moderate the relationship between PSQI and cognitive domain scores (all interactions *p* > 0.2).

*Post hoc* analyses examining the relationships between specific sleep components and sustained attention revealed significant correlations between sustained attention and subjective sleep quality as well as sleep disturbance. The group reporting good sleep quality (n = 174) showed significantly better performance in sustained attention compared with the group with bad sleep quality (n = 29) [F(1,197) = 12.61, *p* < 0.0001, Cohen’s d ES = 0.72] (Supplement 1). The group with less sleep disturbance (n = 149) performed better than the group with more sleep disturbance (n = 54) [F(1,197) = 6.83, *p* < 0.009, ES = 0.42]. No significant associations between sustained attention and the other PSQI components were found.

### PSQI and subjective memory complaints

**Figure 2.**
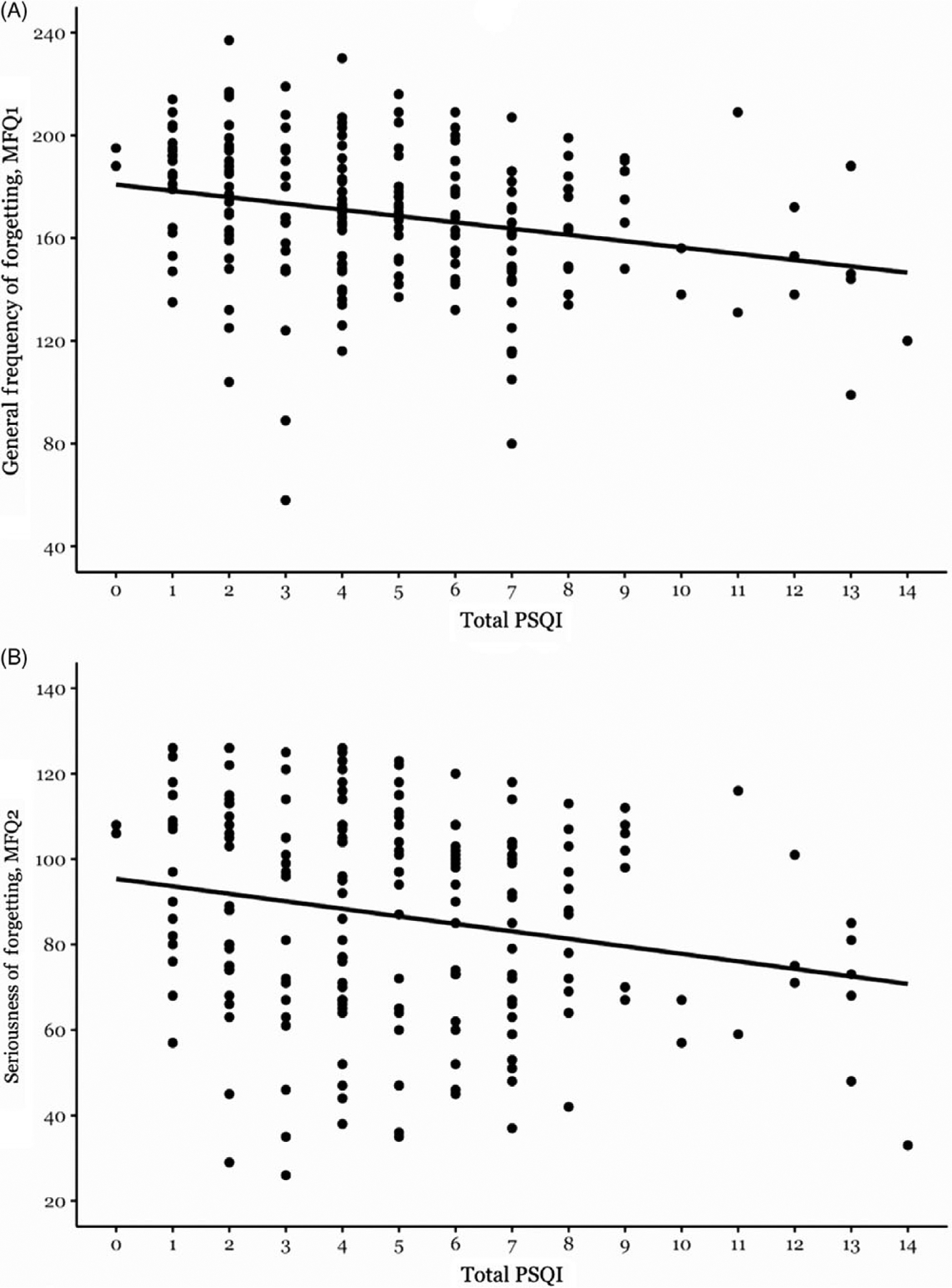
(A) Association between Total PSQI and general frequency of forgetting (MFQ1) and (B) association between Total PSQI and seriousness of forgetting (MFQ2).

Total PSQI was significantly associated with both general frequency of forgetting (MFQ1) [F(1,195) = 12.11, *p* = 0.001, partial r = − 0.24 (Figure 2A)] and seriousness of forgetting (MFQ2), [F(1,195) = 9.95, *p* = 0.002, partial r = − 0.22 (Figure 2B)] (Table 3). None of the interaction terms of PSQI total with age, gender, ApoE4, and OSA risk reached significance.

Sleep latency and daytime sleepiness were significantly associated with MFQ1 and MFQ2. Participants with sleep latency ≤ 15 minutes (n = 87) reported significantly less frequency of forgetting [F(1,195) = 8.7, *p* = 0.004, ES = 0.42] and less seriousness of forgetting [F(1,195) = 12.95, *p* < 0.001, ES = 0.52] compared to participants with sleep latency > 15 minutes (n = 116). Compared to the group who reported daytime sleepiness (n = 106), the group with no daytime sleepiness (N = 97) had less frequency of forgetting [F(1,195) = 12.69, *p* < 0.001, ES = 0.51] and less seriousness of forgetting [F(1,195) = 7.87, *p* = 0.006, ES = 0.40]. Lastly, the group with less sleep disturbance (n = 149) reported less seriousness of forgetting [F(1,195) = 6.53, *p* = 0.011, ES = 0.41] (Supplement 2).

## Discussion

In this cross-sectional study of a large sample of non-demented older adults, we found that poorer sleep quality, specifically fragmented or disrupted sleep, was significantly associated with worse performance in sustained attention and increased memory complaints, and not related to simple attention and memory recall. While there was an association of sleep concerns with poorer executive function, this association did not survive the statistical correction for multiple comparisons. The ES of the significant associations ranged from 0.4 to 0.7, indicating that they were medium to large effects and thus clinically meaningful. The sample of older adults recruited for this study had mild memory complaints, putting them at increased risk for future cognitive decline; therefore, this finding of an association between sleep and cognition in an at-risk sample is important and may serve to inform future preventive interventions that can target sleep to see if they improve cognitive functioning as well in at-risk older adults.

This association of poorer sleep and a reduction in sustained attention is consistent with a previous study indicating that as little as one night of sleep fragmentation is correlated with impaired sustained attention (Martin *et al*., 1996). In particular, we found that performance in sustained attention measures was strongly associated with self-reported sleep quality (with a large ES of 0.7). Maintaining sustained attention involves a complex interplay of several brain regions and poor sleep quality has been associated with an increased rate of atrophy across frontal, temporal, and parietal regions (Sexton *et al*., 2014). Thus while the current findings are insufficient to postulate an exact mechanism of why poorer sleep and sustained attention may be related, it is plausible that the widespread cortical atrophy associated with sleep quality may be a cause of worse performance in sustained attention.

By contrast, self-reported sleep quality was not significantly associated with simple attention and memory. While there was an association between poorer sleep quality and worse executive function, this did not survive correction. In the present study, we measured executive functioning using two components: set-shifting and inhibition (Miyake *et al*., 2000). Previous studies have shown significant associations between set-shifting and sleep quality (Blackwell *et al*., 2006; 2011; Yaffe *et al*., 2007). Moreover, in a study of 157 healthy older adults, reports of poorer sleep quality were significantly associated with worse performance on tasks of working memory and set-shifting compared to reports of better sleep quality (Nebes *et al*., 2009). The discrepancy in findings among studies could be related to the younger age of the participants included in our study, who were on average more than 10 years younger than those in the other studies. In addition, while Blackwell *et al*. (2011) and Nebes *et al*. (2009) used the PSQI as a subjective sleep quality measure, Blackwell *et al*. (2006) used objective sleep measurements derived from actigraphy.

We found that subjective perceptions of memory functioning, both general frequency and seriousness of forgetting, were significantly linked to the PSQI total score, sleep latency, and daytime sleepiness. Such associations have been previously reported in older African American and Korean adults, where lengthened sleep latency was associated with severe subjective memory complaints (Gamaldo *et al*., 2019; Kang *et al*., 2017). Thus, adults with frequent memory complaints tend to report longer periods to fall asleep, or decreased sleep efficiency. Subjective memory problems also have been shown to be an early indicator of subsequent cognitive decline in older individuals (Gifford *et al*., 2014; Reisberg *et al*., 2010). Thus, our finding that sleep disturbances, even in participants with only a mild degree of self-reported sleep problems, were associated with a greater degree of subjective memory complaints is noteworthy. It is possible that those reporting poor sleep are at increased risk for future cognitive decline, and vice versa.

A strength of our study is that we assessed a broad set of neurocognitive measures in a relatively large sample, but some potential limitations need consideration when interpreting the results. Sleep quality was not evaluated using objective measures such as polysomnography or actigraphy. Examining brain activity through different sleep stages may provide more insight in terms of neurophysiological mechanisms of how sleep affects the different domains of cognitive function. Sleep quality was measured using self-reports, a common and convenient method, and previous research indicates that PSQI self-report measures correlate with objective measures of sleep (e.g. Bliwise *et al*., 2014). However, not all investigations show significant correlations between the PSQI self-report measure and objective sleep measures in both younger and older adults (Grandner *et al*., 2006). Thus, the PSQI may not be the best sleep quality measure for older adults, due to its reliance on participant’s recall capacity (Landry *et al*., 2015). In addition, PSQI questions were designed to track sleep quality within a 1-month period (Backhaus *et al*., 2002) and can measure various patterns of sleep problems; however, insomnia or other sleep disorders that can affect cognition cannot be diagnosed using this assessment tool. Also, we analyzed components of sleep using categories, and may have thus not been able to detect some nuances in associations with cognition and memory self-reports; for example, not only short sleep, but long sleep (e.g. >9 hours) can also be associated with worse cognitive functions and the ability to detect such nonlinear relationships may have been lost by dichotomizing sleep duration. Due to the lack of available data, we were also unable to examine sleep duration as a continuous variable or examine the factor scores of PSQI in addition to the PSQI component scores.

## Supplementary Material

supplemental information

## Figures and Tables

**Table 1.** Baseline demographic and clinicalcharacteristics

CHARACTERISTICS		MEAN (SD)/n (%)
*Demographics*		
Age (years)		60.4 (6.5)
Gender^a^	Female	140 (69.0)
Ethnicity^a^	Caucasian	141 (69.5)
	Asian	22 (10.8)
	Hispanic	20 (9.9)
	Black	15 (7.4)
Marital status^a^	Married	119 (58.6)
Education (years)		16.4 (2.1)
ApoE4 status^a^(n = 189)	E4 allele carrier	46 (24.3)
*Clinical measures*		
Montreal Cognitive Assessment		27.7 (1.5)
Beck Depression Inventory^b^		4 (0–18)
STOP-BANG questionnaire (sleep apnea risk)^a^	High risk of sleep apnea	52 (25.6)
Total Pittsburgh Sleep Quality Index (PSQI)		4.9 (2.9)
PSQI component 1: Subjective sleep quality^a^	Bad quality	29 (14.3)
PSQI component 2: Sleep latency^a^	>15 minutes	116 (57.1)
PSQI component 3: Sleep duration^a^	≤ 7 hours	94 (46.3)
PSQI component 4: Habitual sleep efficiency^a^	≤ 85%	53 (26.1)
PSQI component 5: Sleep disturbances^a^	More disturbances	54 (26.6)
PSQI component 6: Use of sleep medication^a^	Used this week	45 (22.2)
PSQI component 7: Daytime dysfunction^a^	Problem with sleepiness	106 (52.2)

a reported as n (%).

b reported as median score (range).

**Table 2.** Neuropsychological measures

Cognitive domains	Measures	Mean (SD)
Simple attention	T-score for detectability (d-prime)	45.3 (11.3)
	T-score for hit reaction time	54.8 (12.2)
	T-score for standard error	49.4 (10.0)
	T-score for reaction time by interstimulus interval	54.2 (9.0)
	T-score for standard error by interstimulus interval	54.4 (11.8)
Sustained attention	T-score for hit reaction time by block	47.8 (9.6)
	T-score for standard error by block	53.8 (8.8)
Executive function^a^	Time on TMT part B - A (seconds)	40.2 (21.0)
	T-score for commission errors	45.1 (7.8)
Memory (Recall)	BVMT-R (delayed score)	8.4 (2.1)
	Buschke Selective Reminding Test (delayed recall)	10.2 (2.1)

Abbreviations: TMT, Trail Making Test; BVMT-R, The Brief Visual Memory Test – Revised.

For the scores in simple attention, sustained attention, and executive function, *lower score* indicates *better performance*.

a Executive function uses the average of three z-scores, time on TMT part B − A, T-score for commission errors, and T-score for hit reaction time.

**Table 3.** Association between Total PSQI and cognitive domain scores/memory self-reports

	Partial r^a^	F-statistics	*p*-value	Slope^b^ (SE)
Cognitive domain^c^				
Simple attention	0.12	3.02	0.084	0.02 (0.01)
Sustained attention	− 0.22	10.03	0.002^d^	− 0.06 (0.02)
Executive function	− 0.16	4.91	0.028	− 0.03 (0.02)
Recall memory	− 0.04	0.33	0.568	− 0.01(0.02)
Memory self-report^e^				
General frequency of forgetting (MFQ1)	− 0.24	12.11	0.001^f^	− 2.34 (0.67)
Seriousness of forgetting (MFQ2)	− 0.22	9.95	0.002^f^	− 1.84 (0.58)

a Adjusted for age, gender, education, and MOCA.

b Unstandardized estimates.

c n = 203; F-statistics are F(1,197).

d Significant result, *p* < 0.0125 (0.05/4).

e n = 201; F-statistics are F(1,195).

f Significant result, *p* < 0.025 (0.05/2).
