# Supplementary material for: Sleep quality, neurocognitive performance, and memory self-appraisal in middle-aged and older adults with memory complaints
Source: Int Psychogeriatr. Author manuscript; Available in PMC 2022 Jul 1. (PMC8004546; doi:10.1017/S1041610220003324)
Supplement: supplemental information [file NIHMS1646021-supplement-supplemental_information.docx]

**Supplement 1. Association between PSQI components and sustained attention z-score**

| PSQI component | Sustained Attention z-score | F(1,197) | p-value | ES |
| --- | --- | --- | --- | --- |
| PSQI Subjective sleep quality |  |  |  |  |
| Good *(n = 174)* | .08 (.06) | 12.61 | .001^a^ | 0.72 |
| Bad *(n = 29)* | -.48 (.15) |  |  |  |
| PSQI Sleep latency |  |  |  |  |
| Less than or equal to 15 min *(n = 87)* | .10 (.09) | 2.28 | .13 | 0.22 |
| More than 15 min *(n = 116)* | -.08 (.08) |  |  |  |
| PSQI Sleep duration |  |  |  |  |
| More than 7 hours *(n = 109)* | -.002 (.08) | .001 | .98 | 0.004 |
| 7 hours or less *(n = 94)* | .002 (.09) |  |  |  |
| PSQI Habitual sleep efficacy |  |  |  |  |
| More than 85% *(n = 150)* | .06 (.07) | 2.98 | .09 | 0.28 |
| 85% or less *(n = 53)* | -.17 (.11) |  |  |  |
| PSQI Sleep disturbance |  |  |  |  |
| Less sleep disturbance *(n = 149)* | .09 (.07) | 6.83 | .009^a^ | 0.42 |
| More sleep disturbance *(n = 54)* | -.25 (.11) |  |  |  |
| PSQI Use of sleep medication |  |  |  |  |
| No use in this week *(n = 158)* | .01 (.07) | .02 | .70 | 0.02 |
| Use in this week *(n = 45)* | -.04 (.12) |  |  |  |
| PSQI Daytime dysfunction |  |  |  |  |
| No daytime dysfunction *(n = 97)* | .14 (.08) | 5.17 | .02 | 0.32 |
| Problem with daytime dysfunction *(n = 106)* | -.125 (.08) |  |  |  |

Adjusted for age, gender, education, and MOCA.

^a^Significant result (p < .0125)

**Supplement 2.**

**Association between PSQI components and General Frequency of Forgetting (MFQ1)**

| PSQI component | MFQ1  mean (SD) | F(1,195) | p-value | ES |
| --- | --- | --- | --- | --- |
| PSQI Subjective sleep quality |  |  |  |  |
| Good *(n = 172)* | 169.33 (28.33) | .53 | .47 | 0.15 |
| Bad *(n = 29)* | 165.17 (28.49) |  |  |  |
| PSQI Sleep latency |  |  |  |  |
| Less than or equal to 15 min *(n = 85)* | 175.53 (27.94) | 8.7 | .004^a^ | 0.42 |
| More than 15 min *(n = 116)* | 163.74 (27.90) |  |  |  |
| PSQI Sleep duration |  |  |  |  |
| More than 7 hours *(n = 107)* | 169.78 (28.45) | .31 | .58 | 0.08 |
| 7 hours or less *(n = 94)* | 167.52 (28.50) |  |  |  |
| PSQI Habitual sleep efficacy |  |  |  |  |
| More than 85% *(n = 148)* | 171.05 (28.35) | 3.66 | .06 | 0.31 |
| 85% or less *(n = 53)* | 162.23 (28.61) |  |  |  |
| PSQI Sleep disturbance |  |  |  |  |
| Less sleep disturbance *(n = 148)* | 171.44 (28.11) | 5.07 | .025 | 0.36 |
| More sleep disturbance *(n = 53)* | 161.15 (28.39) |  |  |  |
| PSQI Use of sleep medication |  |  |  |  |
| No use in this week *(n = 157)* | 167.78 (28.44) | .78 | .38 | 0.15 |
| Use in this week *(n = 44)* | 172.12 (28.66) |  |  |  |
| PSQI Daytime dysfunction |  |  |  |  |
| No daytime dysfunction *(n = 95)* | 176.07 (27.58) | 12.69 | <.001^a^ | 0.51 |
| Problem with daytime dysfunction *(n = 106)* | 162.15 (27.59) |  |  |  |

Adjusted for age, gender, education, and MOCA.

^a^Significant results (p < .025).

**Association between PSQI components and Seriousness of Forgetting (MFQ2)**

| PSQI component | MFQ2  mean (SD) | F(1,195) | p-value | ES |
| --- | --- | --- | --- | --- |
| PSQI Subjective sleep quality |  |  |  |  |
| Good *(n = 172)* | 87.38 (24.52) | .90 | .35 | 0.19 |
| Bad *(n = 29)* | 82.7 (24.56) |  |  |  |
| PSQI Sleep latency |  |  |  |  |
| Less than or equal to 15 min *(n = 85)* | 93.8 (23.88) | 12.95 | <.001^a^ | 0.52 |
| More than 15 min *(n = 116)* | 81.5 (23.81) |  |  |  |
| PSQI Sleep duration |  |  |  |  |
| More than 7 hours *(n = 107)* | 88.77 (24.52) | 1.62 | .20 | 0.18 |
| 7 hours or less *(n = 94)* | 84.35 (24.53) |  |  |  |
| PSQI Habitual sleep efficacy |  |  |  |  |
| More than 85% *(n = 148)* | 87.12 (24.7) | .16 | .69 | 0.06 |
| 85% or less *(n = 53)* | 85.52 (24.97) |  |  |  |
| PSQI Sleep disturbance |  |  |  |  |
| Less sleep disturbance *(n = 148)* | 89.35 (24.21) | 6.53 | .011^a^ | 0.41 |
| More sleep disturbance *(n = 53)* | 79.3 (24.46) |  |  |  |
| PSQI Use of sleep medication |  |  |  |  |
| No use in this week *(n = 157)* | 86.11 (24.56) | .41 | .52 | 0.11 |
| Use in this week *(n = 44)* | 88.83 (24.81) |  |  |  |
| PSQI Daytime dysfunction |  |  |  |  |
| No daytime dysfunction *(n = 95)* | 91.76 (24.07) | 7.87 | .006^a^ | 0.40 |
| Problem with daytime dysfunction *(n = 106)* | 82.17 (24.09) |  |  |  |

Adjusted for age, gender, education, and MOCA. ^a^Significant results (p < .025).
